# HELPING AGING VETERANS PLAN AHEAD (HAVE)

**DOI:** 10.1093/geroni/igae098.3995

**Published:** 2024-12-31

**Authors:** Dennis Sullivan, Bonnie Dawson, Angela McFadden, Ramona Rhodes, Linda Sawyer, Christina Crawford

**Affiliations:** Central Arkansas Veterans Healthcare System, North Little Rock, Arkansas, United States; Department of Veterans Affairs, North Little Rock, Arkansas, United States; Department of Veterans Affairs, North Little Rock, Arkansas, United States; Department of Veterans Affairs, North Little Rock, Arkansas, United States; Department of Veterans Affairs, North Little Rock, Arkansas, United States; Central Arkansas Veterans Healthcare System, North Little Rock, Arkansas, United States

## Abstract

It is estimated that up to 60% of older Veterans (OVs) do not have an Advance Directive (AD). The HAVE clinical program initiative was developed to address this problem by helping OVs understand the importance of having an ACP. Initially, OVs at high risk for institutionalization (per Office of GEC PLI List) were invited (by letter and phone call) to participate in HAVE. At the first visit (conducted in-person or by VVC), the OV (and Caregiver) met with a HAVE lay health advisor (HLHA) who conducted a baseline assessment including administration of Brief Health Literacy, Knowledge of Care options, and Quality of Life rating questionnaires. Education focused on incorrect responses was provided and AD materials were provided for home review. The HLHA then used motivational interviewing techniques to help the OV articulate his/her desires for ACP and to indicate what he/she thought was important that he/she could do to ensure these wishes were honored and to then create SMART goals for doing this. Up to three follow-up phone visits were conducted. In the first four months of the program, 218 eligible OVs were identified. Of these, 17 refused or could not be contacted, 40 already had an AD, and 55 were scheduled for a first visit. To date, 16 of the 17 OVs completing the HAVE program created a new AD and 6 OVs updated their older AD. Given the early success of HAVE, the program is being expanded to Veterans over age 60.

